# Biallelic mutations in *NRROS* cause an early onset lethal microgliopathy

**DOI:** 10.1007/s00401-020-02137-7

**Published:** 2020-02-25

**Authors:** Colin Smith, Barry W. McColl, Anirudh Patir, Jack Barrington, Jeremy Armishaw, Antonia Clarke, Jenny Eaton, Vivienne Hobbs, Sahar Mansour, Melinda Nolan, Gillian I. Rice, Mathieu P. Rodero, Luis Seabra, Carolina Uggenti, John H. Livingston, Leslie R. Bridges, Iona J. M. Jeffrey, Yanick J. Crow

**Affiliations:** 1 Academic Department of Neuropathology, Centre for Clinical Brain Sciences, University of Edinburgh, Edinburgh, UK; 2 Centre for Discovery Brain Sciences, UK Dementia Research Institute, University of Edinburgh, Edinburgh, UK; 3 The Roslin Institute, University of Edinburgh, Edinburgh, UK; 4 Department of Paediatrics, Bay of Plenty District Health Board, Tauranga Hospital, Tauranga, New Zealand; 5 Paediatric Neurology Department, St Georges Healthcare NHS Trust, London, UK; 6 Genetic Health Service New Zealand, Auckland District Health Board, Auckland City Hospital, Auckland, New Zealand; 7 Department of Clinical Genetics, SW Thames Regional Genetics Service, St George’s Hospital, University of London, London, UK; 8 Department of Paediatric Neurology, Starship Children’s Health, Auckland, New Zealand; 9 Division of Evolution and Genomic Sciences, School of Biological Sciences, Faculty of Biology, Medicine and Health, University of Manchester, Manchester Academic Health Science Centre, Manchester, UK; 10 Laboratory of Neurogenetics and Neuroinflammation, Institut Imagine, Paris, France; 11 Centre for Genomic and Experimental Medicine, MRC Institute of Genetics and Molecular Medicine, University of Edinburgh, Edinburgh, UK; 12 Department of Paediatric Neurology, Leeds General Infirmary, Leeds, UK; 13 Department of Cellular Pathology, St George’s University Hospitals NHS Foundation Trust, London, UK

Microglia are tissue-resident macrophages playing essential roles in central nervous system (CNS) development and homeostasis [14, 17]. The importance of microglia for brain health in humans has been highlighted by the definition of Mendelian disorders associated with dysfunction of microglia-related proteins. These so-called microgliopathies [20] comprise a diverse set of neurological phenotypes including disease due to mutations in *CSF1R* [5, 8, 13], *DAP12* and *TYROBP/TREM2* [4, 7], *USP18* [3, 11, 16, 18], and *IRF8* [2, 6]. Here, we describe a novel early onset lethal encephalopathy due to mutations in the microglial-associated protein NRROS.

We ascertained three patients demonstrating a stereotyped clinical and neuroradiological phenotype (Supplementary material). Patients 1 (P1) and P2, both females, were the first and third children of non-consanguineous parents of Maori descent (family F1442), whilst P3 (family F2382), a male, was the first child of first cousin south Asian parents (Supplementary Figure 1). All three children were born after a normal pregnancy and delivery, and early development was unremarkable. However, in the second year of life, they experienced the onset of refractory seizures and neurodegeneration, leading to death between the ages of 27 and 36 months. Metabolic testing, including for mitochondrial dysfunction, was non-contributory. Neuroimaging initially demonstrated fine calcification at the depths of the cerebral gyri, with normal white matter (Fig. 1). As disease progressed, repeat imaging revealed increased calcification, severe generalized atrophy with ventricular dilatation, and diffuse signal changes in cerebral and cerebellar white matter.

**Fig. 1:**
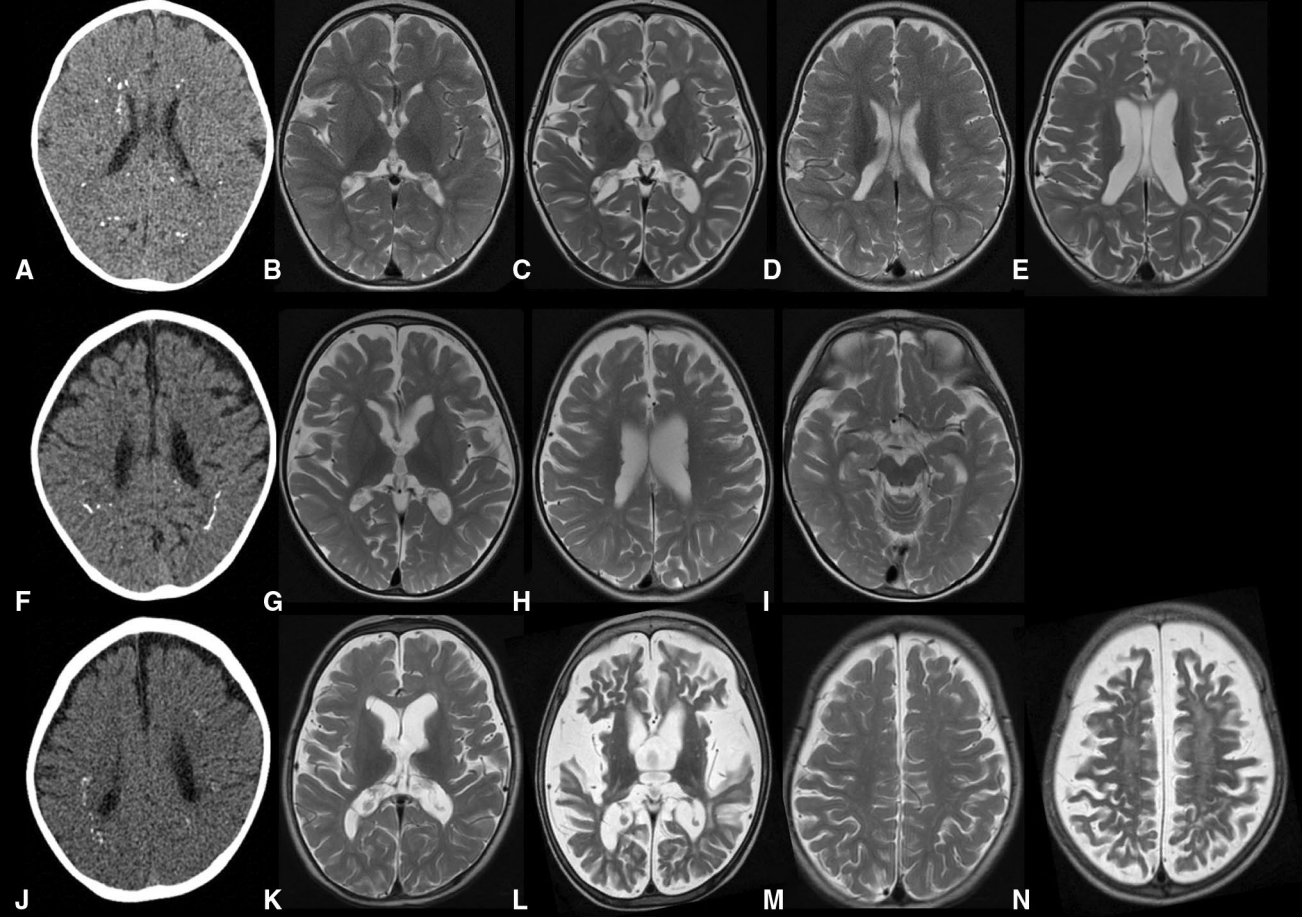
Neuroimaging of patients from F1442 and F2382. CT images of patient 1 (P1) aged 9 months (**a**), patient 2 (P2) aged 17 months (**f**), and patient 3 (P3) aged 14 months (**j**) demonstrate widespread spot and linear calcification in the deep and sub-cortical white matter. T2-weighted axial MR images of P1 aged 9 months (**b**, **d**) and 27 months (**c**, **e**), P2 aged 17 months (**g**, **h**, **i**), and P3 aged 14 months (**k**, **m**) and 27 months (**l**, **n**). Initial imaging shows mild cerebral volume loss with relatively good myelination. Follow-up shows rapidly progressive cortical and sub-cortical atrophy in P1 and P3. There is diffuse high signal in the white matter in P3 (**l**, **n**), but not P1

Exome sequencing identified homozygous *NRROS* variants in the affected children from both families: a c.1777C > T/p.(Gln593*) and a c.1257del/p.(Gly420AlafsTer14) in F1442 and F2382, respectively. Cellular material was not available from any of the patients. However, both of these variants are predicted to result in a truncated protein, and both are very rare, with the p.(Gln593*) not previously recorded, and the p.(Gly420AlafsTer14) reported on only 1 of 251,438 alleles on gnomAD.

Detailed pathological examination was undertaken on P3, demonstrating abnormalities confined to the CNS. Gross examination indicated a significant cerebral atrophy (Supplementary Figure 2). Histologically, there was both grey and white matter pathology throughout the cerebrum, cerebellum, and brainstem. Focal calcification was noted in the neuropil. There was widespread neuronal loss with reactive gliosis throughout the grey matter (Supplementary Figure 3). The most striking pathological finding was the accumulation of foamy macrophages, predominantly in a perivascular distribution, throughout the white matter, extending from frontal to occipital white matter (Fig. 2a–c), and through cerebellar white matter and descending corticospinal pathways in the basis pontis. These foamy cells immunoreacted with CD68, MHC Class II (CR3/43), and p22phox (Fig. 2c–f), but did not immunoreact with CD163, Iba1, NRROS, CD3, P2Y12, or TMEM119 (Supplementary Figure 3). There was reduced myelin basic protein (MBP) expression in the white matter (Fig. 2g) compared to age-matched controls, although there was preservation of U fibers. Occasional axonal spheroids were noted, albeit this was not a prominent feature (Supplementary Figure 3).

**Fig. 2:**
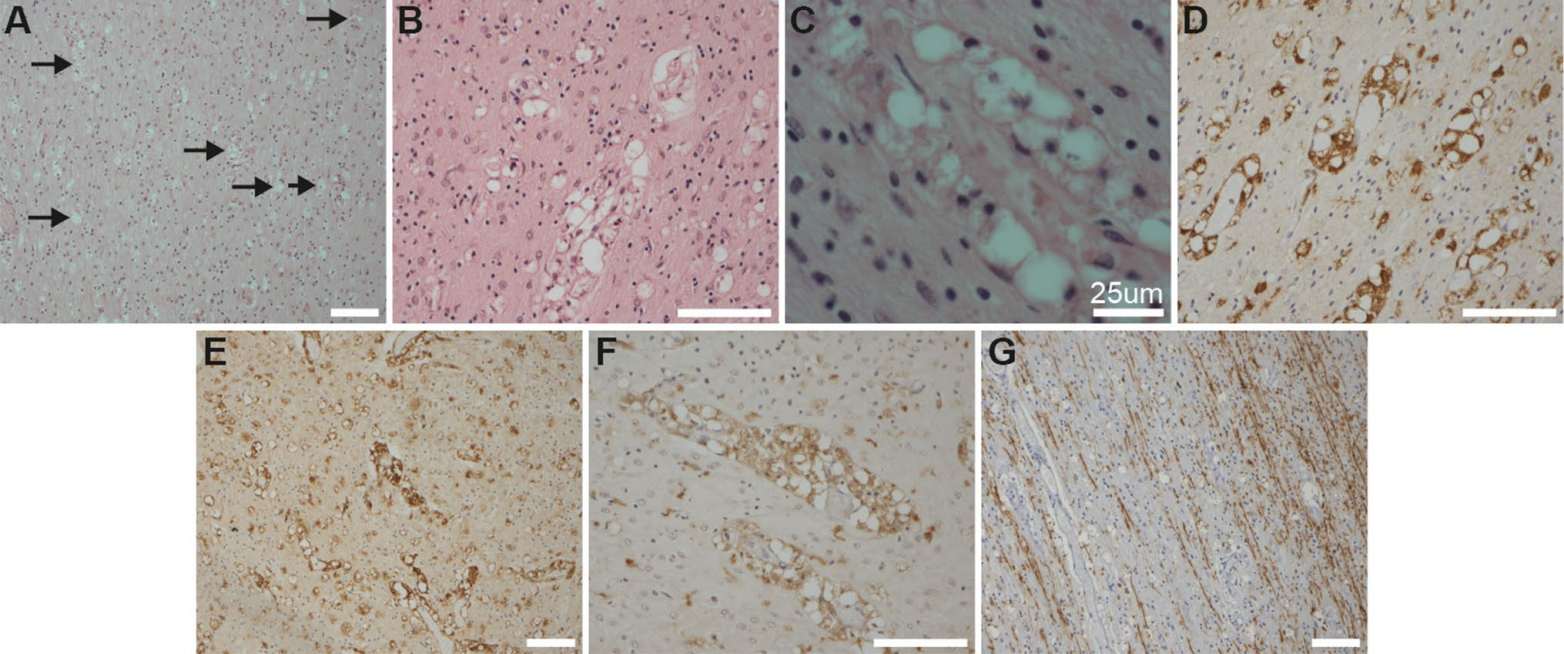
Post-mortem histological examination of patient 3 (P3). **a** Low-power view of occipital white matter with accumulation of foamy cells, predominantly in a perivascular location (black arrows for highlighted examples) (H&E: × 100); **b** the perivascular distribution being highlighted at a higher power (H&E × 200). Granular material is present in the cytoplasm of these foamy cells (**c** H&E × 600). The white matter foamy cells were predominantly, but not exclusively, limited to a perivascular distribution and expressed CD68 (**d** CD68 IHC × 200), HLA DR/DP/DQ (**e** CR3/43 IHC × 100), and p22PHOX (**f** p22phox IHC × 200). There was partial preservation of myelin in sub-cortical U fibers, but loss of myelin when assessed by myelin basic protein expression in deeper white matter (**g** myelin basic protein IHC × 100)

We assessed the cellular expression of *NRROS*, and the mouse homolog *Nrros* (*Lrrc33*), in human and mouse brain respectively, by mining curated transcriptomic data sets (Supplementary Figure 4). In fresh post-mortem human cortical microglia and brain samples, *NRROS* was highly expressed in isolated microglia, although less abundantly than established microglial signature genes. The expression of *NRROS* was enriched > 50-fold in microglia compared to whole brain, indicating that microglia are the major cell type expressing *NRROS* in human brain parenchyma. A similar pattern of highly enriched expression of *Lrrc33/Nrros* was observed in CD11b^+^ microglia/macrophages in mouse brain relative to brain extracts, and in microglia versus other parenchymal cell types. Comparison of parenchymal microglia with CNS perivascular macrophages (PVMs) showed significantly greater expression in the latter.

The clinical features observed in our patients recapitulate those in mice with *Nrros/Lrrc33* deficiency. *Nrros−*/−mice exhibited progressive neurological decline, including motor defects and abnormal locomotor activity, from age 2–3 months and death by 6 months of age [15, 21]. Neuropathology in these mouse models includes neuronal loss, demyelination, axonal pathology, astrogliosis, and the increased presence of foamy macrophages, all of which were seen in our case. Of note, there was no indication of immune-mediated inflammation in our case or either of these mouse models.

NRROS is a leucine-rich repeat containing transmembrane protein localized to the endoplasmic reticulum, and preferentially expressed in myeloid cells. Reported functions include the regulation of reactive oxygen species (ROS) production through control of NOX2 stability [12], responsiveness of Toll-like receptor signaling [19], and processing/activation of transforming growth factor (TGF)-β via physical interactions with the latent complex [10, 15]. NRROS expression is restricted to microglia within the CNS parenchymal compartment in humans and mice. The present case showed disruption to the distribution, density, and cell morphology of IBA1 cells alongside loss of P2Y12 staining and weak TMEM119 immunoreactivity, indicative of marked parenchymal microglial abnormalities. Both *Nrros−*/− mouse studies observed a loss of homeostatic gene expression profile which included suppression of *P2ry12* and *Tmem119* expression, and a shift towards a phenotype resembling PVMs [15, 21]. Although *Nrros* is expressed in peripheral mononuclear cells, a series of crosses and bone marrow transplant experiments showed a negligible contribution of peripheral macrophages to the onset of the *Nrros−*/− phenotype [21]. Of note, selective deletion of *Nrros* in microglia during pregnancy indicated a cell-intrinsic role for NRROS. In contrast, *Nrros* deletion induced in 3-week-old mice did not cause neuropathological changes or neurological abnormalities [21], implying that NRROS is important during microglial establishment at embryonic/postnatal stages, but may be dispensable for maintenance of adult microglia. Functions of NRROS proposed above, notably in ROS and TGFβ regulation, may be important in disease pathogenesis. p22phox was markedly up-regulated in PVMs in our case, suggesting that an absence of functional NRROS may result in increased p22phox-NOX2 binding, with the potential for increased superoxide radical formation. However, a cross of *Nrros−*/− and *Cybb−*/− (encoding NOX2) mice did not rescue the *Nrros−*/− phenotype [21]. Mice with CNS or microglial-restricted disruption during development of other key nodes in the TGFβ activation/signaling pathway, including deletion of αVβ8 integrin or TGFBR2 [1], develop highly similar pathological, microglial, and neurological abnormalities to *Nrros−*/− mice. Moreover, human TGFβ1 loss-of-function mutations causing early onset leukoencephalopathy were described recently [9].

Taken together with the mouse data, our findings indicate that NRROS is indispensable in controlling the early development of a homeostatic microglial population and/or its ongoing preservation in the postnatal brain, thereby suggesting a loss of NRROS function as a novel microgliopathy in humans.

## Electronic supplementary material

Below is the link to the electronic supplementary material.

Supplementary file1 (PDF 5202 kb)
