# Supplementary material for: Biallelic mutations in NRROS cause an early onset lethal microgliopathy
Source: Acta Neuropathol. 2020 Feb 25;139(5):947–51. doi: 10.1007/s00401-020-02137-7 (PMC7181551; doi:10.1007/s00401-020-02137-7)
Supplement: Supplementary file 1 — Supplementary file1 (PDF 5202 kb) [file 401_2020_2137_MOESM1_ESM.pdf]

BIALLELIC MUTATIONS IN *NRROS* CAUSE AN EARLY ONSET LETHAL MICROGLIOPATHY

MATERIALS AND METHODS SUPPLEMENT

Patient recruitment and DNA sampling

Sample collection was performed with written informed consent, and DNA storage and usage was approved by the Comité de Protection des Personnes (ID-RCB/EUDRACT: 2014-A01017-40) in France, the Leeds-west Multicentre Research Ethics Committee (REC reference: 10/H1307/2; IRAS project ID: 62971), and Cambridge South REC (approval: 10/H0305/83, through the Deciphering Developmental Disorders study - DDD). The age-matched control tissue was from a 2-year old with a cardiac anomaly accessed from the Edinburgh Brain bank (ethical approval LR/16/ES/0084).

Sequencing

In F1442, DNA was extracted from whole blood samples using standard methods. Whole-exome sequencing was performed in the Crow laboratory using genomic DNA from the two affected individuals, the SureSelect Human All Exon kit (Agilent Technologies) for targeted enrichment and Illumina HiSeq2000 for sequencing. Variants were assessed using the *in silico* programs SIFT (http://sift.jcvi.org) and Polyphen2 (http://genetics.bwh.harvard.edu/pph2/), and population allele frequencies obtained from the gnomAD (http://gnomad.broadinstitute.org) database. The homozygous variant in *NRROS* was verified by standard Sanger sequencing in both affected individuals and their parents (primers available on request). Mutations in F2382 were identified through data derived from 4,293 trios who underwent exome sequencing as part of the Deciphering Developmental Disorders (DDD) study [1].

Gene expression analysis in human and mouse brain

We surveyed expression of *NRROS* and the mouse orthologue *Lrrc33/Nrros* by mining our own recently generated transcriptomic datasets and those of other groups. Specifically, human *NRROS* expression was assessed using the RNA sequencing dataset of Galatro et al. [3], and mouse *Lrrc/Nrros* expression from Grabert et al. (microarray) [4], Zhang et al. (RNA sequencing) [6] and Zeisel et al. (single cell RNA sequencing) [5]. Data from the first three datasets were extracted from the original source files (available at NCBI GEO) and from the Zeisel dataset using the Myeloid Landscape portal (http://research-pub.gene.com/BrainMyeloidLandscape/#). Technical details of extraction and processing of samples from which the data presented here are derived are available in the original publications.

Clinical description

Patient 1 (P1) and patient 2 (P2), both females, were the first and third children of non-consanguineous parents of Maori descent (family F1442) (Supplementary Fig. 1a). P1 was born after a normal pregnancy and delivery. She presented with a first seizure at age 6 months, which was tonic-clonic and relatively easily controlled. Her development was initially considered to be within normal limits. However, by age 18 months her seizure frequency increased, her seizures became drug resistant and she had clearly lost skills. At her best, aged around 15 months, she was able to sit, grasp toys and was beginning to walk with assistance. Over time she deteriorated progressively with severe spasticity and little interaction, and she subsequently died aged 3 years. Routine karyotyping and extensive metabolic testing, including for mitochondrial dysfunction, was normal. Head circumference was on the 98th percentile at birth, falling to the 10th percentile at the time of her death.

As for her sister, after an unremarkable pregnancy and delivery, P2 initially demonstrated a normal developmental profile, but by age 18 months she had begun to lose skills. Aged 2 years 5 months, P2 died of respiratory failure on a background of severe neurological regression, having lost all of her acquired developmental skills. She also experienced tonic clonic seizures, and demonstrated a fall in her head circumference from the 50th to the 10th percentile.

Patient 3 (P3: family F2382), a male, was the first child of first cousin south Asian parents (Supplementary Fig. 1b). He was born at 39 weeks gestation following a normal pregnancy with normal antenatal scans, weighing 2.5 kg. He was cruising around furniture at age 14 months, at which point there were no developmental concerns. At this point he experienced seizures for the first time, and began to regress thereafter. By age 23 months he could no longer sit, and was admitted to hospital with refractory seizures. Extensive investigations, including respiratory chain analysis of a muscle biopsy, were normal, and he died at the age of 27 months.

Pathological examination

Macroscopic examination
Detailed pathological examination of P3 demonstrated abnormalities confined to the CNS. The head circumference was 45.8cm (-3 SD). The brain was noted to be atrophic (Supplementary Fig. 2a), weighing 534 grams (mean brain weight at 27 months approximately 1120 grams [2]). On sectioning there was generalized cortical atrophy with ventricular enlargement, and the white matter appeared shrunken and grey with focal cystic degeneration (Supplementary Fig. 2b), although with preservation of U-fibers (Supplementary Fig. 2c). The corpus callosum was thinned and there was atrophy of the brainstem.

Supplementary figures

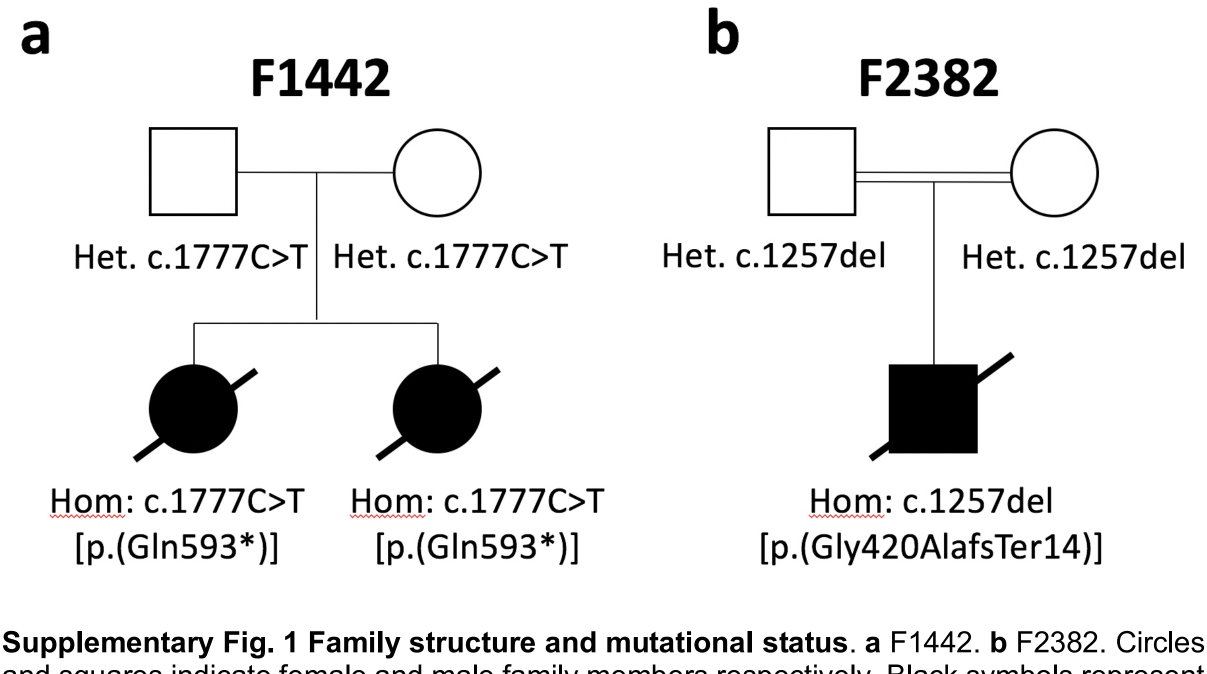

Supplementary Fig. 1 Family structure and mutational status. **a** F1442. **b** F2382. Circles and squares indicate female and male family members respectively. Black symbols represent affected individuals. Diagonal line indicates deceased status. Double line indicates consanguinity (first cousins in this case). Het = Heterozygous; Hom = Homozygous

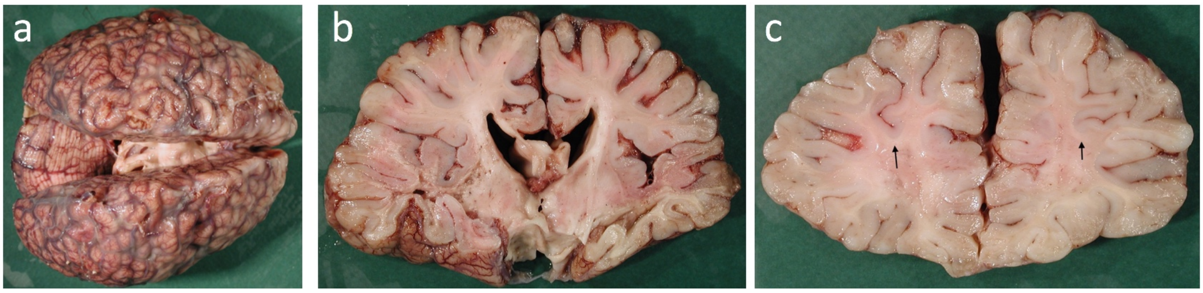

Supplementary Fig. 2 Post-mortem examination of patient 3 (P3). **2a** External view (vertex) of post-mortem brain showing bilateral, symmetrical atrophy. **2b** Coronal section of brain at level of mammillary bodies demonstrating generalized cortical atrophy with reduced white matter, enlarged ventricles and striking cystic degeneration of white matter in both temporal lobes. **2c** Coronal section of brain through the frontal lobes, the white matter showing grey discoloration, with preservation of U-fibers (arrows)

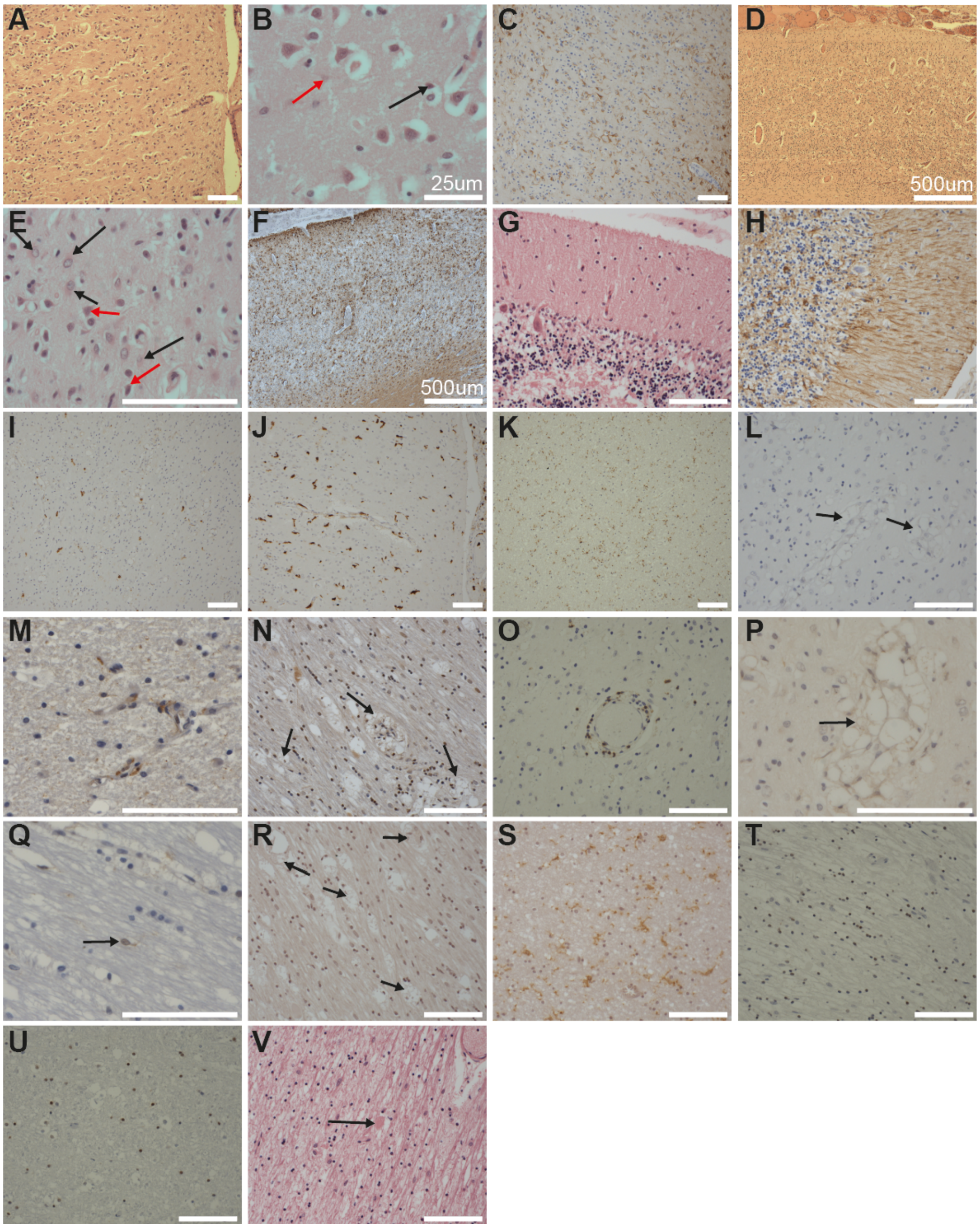

Supplementary Fig. 3 Histological examination of brain of patient 3 (P3). Neuronal loss and reactive gliosis was seen extending from frontal cortex (a: H&E x100; b: H&E x600; c: GFAP IHC x100) to the occipital cortex (d: H&E x40; e: H&E x600; f: GFAP IHC x40). In Supp. fig. 3b from the frontal cortex, a neuron is highlighted by a black arrow, and a ghost outline of a neuron by a red arrow. In Supp. fig 3e from the occipital cortex, examples of reactive astrocytes are identified by black arrows, and a neuron by a red arrow. Purkinje cell loss (g: H&E x200) and reactive Bergmann gliosis was seen in the cerebellar cortex (h: GFAP IHC x200). The perivascular foamy cells showed no expression of Iba1, although cells expressing Iba1 were seen in both white (i: iba1 IHC x100) and grey matter (j: iba1 IHC x100). However, Iba1 immunoreactive cells were greatly reduced in number when compared to age-matched control cases (k; iba1 IHC x100), showing shortened blunted processes. CD163 did not immunoreact with the perivascular cells, highlighted by black arrows (l: CD163 IHC x200), although occasional perivascular CD163 imunoreactive cells were seen in age-matched control (m: CD163 IHC x400). There was no expression of NRROS when assessed by immunohistochemistry, including assessment of the perivascular foamy cells, some of which are highlighted by black arrows (n: NRROS IHC x400). Perivascular CD3 expression was seen, although was not prominent (o: CD3 IHC x200). TMEM119 showed only weak non-specific staining in relation the foamy cells, indicated by the black arrow (p: TMEM119 IHC x400), and showed only very focal immunoreactivity [black arrow] in control white matter (q: TMEM119 IHC x400). P2Y12 showed no expression in foamy cells, indicated by black arrows, (r: P2Y12 IHC x200) when compared to an age-matched control (s: P2Y12 IHC x200). Olig2 was expressed at a similar level in the NRROS mutated case (t: Olig2 IHC x200) when compared to control cases (u: Olig2 IHC x200). Axonal spheroids were seen, but were few in number [black arrow] (v: H&E x200).

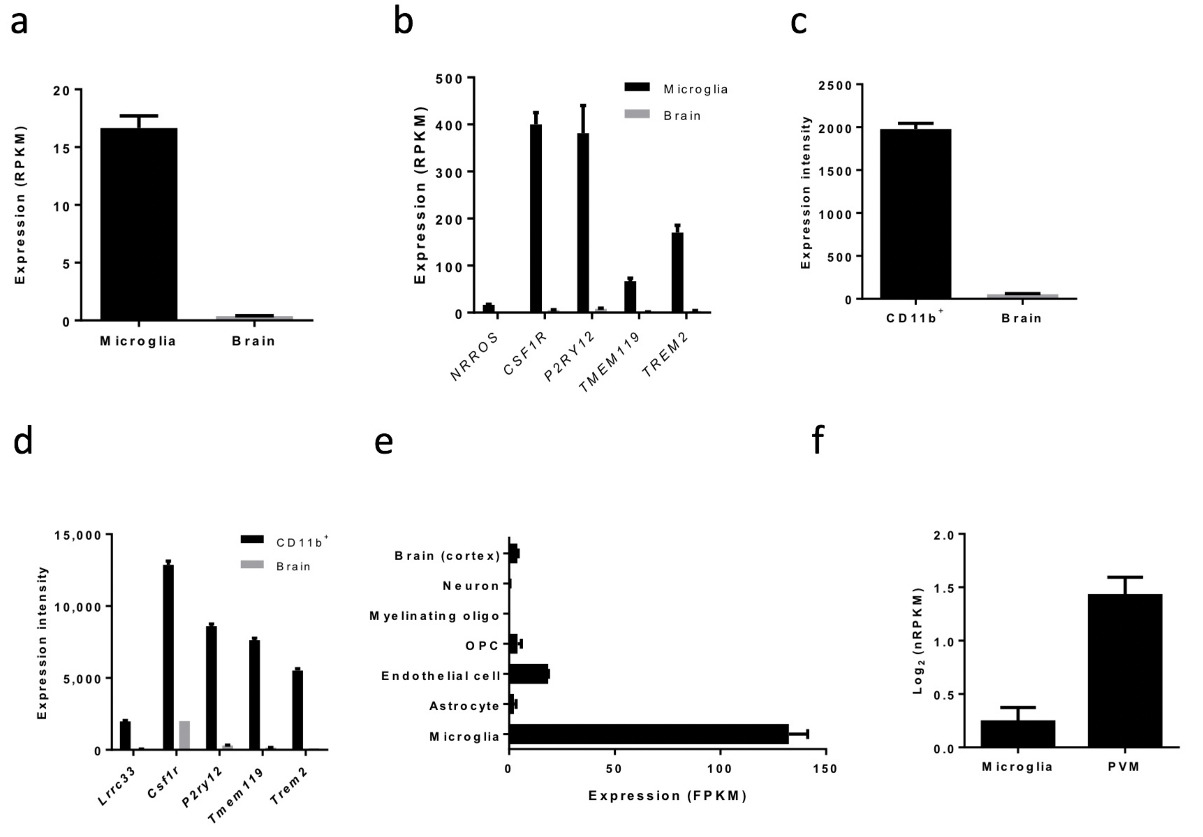

Supplementary Fig. 4 Cellular expression of *NRROS* / *Nrros* (*Lrrc33*) in human and mouse brain. **a** *NRROS* expression in isolated human cortical microglia and cortical whole brain extracts. **b** NRROS expression relative to established highly enriched microglial genes

in isolated human cortical microglia. Data mined from Galatro et al. [3]. **c** *Lrrc33/Nrros* expression in isolated mouse cortical $CD11b^{+}$ sorted cells (i.e. microglia/CNS-associated macs) and cortical brain extracts. **d** *Lrrc33/Nrros* expression relative to established highly enriched microglial genes in isolated mouse cortical $CD11b^{+}$ sorted cells. Data mined from Grabert et al. [4]. **e** *Lrrc33/Nrros* expression in isolated mouse microglia relative to other CNS cell types and brain. Data mined from Zhang et al. [6]. **f** *Lrrc33/Nrros* expression in mouse microglia and perivascular macrophages identified from single-cell RNA sequencing. Data derived from Zeisel et al. [5]. Data show mean ± SEM

Supplementary table

Supplementary table 1: Antibodies used in the neuropathological assessment

	Company	Clone	Dilution	Pre-treatment
**CD68**	Agilent	PG-M1	1:100	Citric
**HLA DR/DP/DQ**	DAKO	CR3/43	1:500	Citric
**Iba1**	ABCAM	EPR16588	1:3000	Citric
**TMEM119**	ABCAM	Cat No: ab185337	1:50	Citric
**P2Y12**	ATLAS	HPA014518	1:500	Tris-EDTA
**CD163**	ABCAM	Cat No: Ab87099	1:1000	Citric
**NRROS**	LifeSpan BioSciences	LRRC33	1:50	Citric
**P22phox**	ABCAM	Cytochrome b245	1:250	Citric
**GFAP**	DAKO	Cat No: Z0334	1:800	None
**CD3**	Leica	Cat No:NCL-L-CD3-565	1:100	
**Olig2**	ABCAM	Cat No: AB109186	1:100	Citric

References

1 Deciphering Developmental Disorders S (2015) Large-scale discovery of novel genetic causes of developmental disorders. Nature 519: 223-228 Doi 10.1038/nature14135

2 Dekaban AS (1978) Changes in brain weights during the span of human life: relation of brain weights to body heights and body weights. Annals of neurology 4: 345-356 Doi 10.1002/ana.410040410

3 Galatro TF, Holtman IR, Lerario AM, Vainchtein ID, Brouwer N, Sola PR, Veras MM, Pereira TF, Leite REP, Moller Tet al (2017) Transcriptomic analysis of purified human cortical microglia reveals age-associated changes. Nat Neurosci 20: 1162-1171 Doi 10.1038/nn.4597

4 Grabert K, Michoel T, Karavolos MH, Clohisey S, Baillie JK, Stevens MP, Freeman TC, Summers KM, McColl BW (2016) Microglial brain region-dependent diversity and selective regional sensitivities to aging. Nat Neurosci 19: 504-516 Doi 10.1038/nn.4222

5 Zeisel A, Munoz-Manchado AB, Codeluppi S, Lonnerberg P, La Manno G, Jureus A, Marques S, Munguba H, He L, Betsholtz Cet al (2015) Brain structure. Cell types in the mouse cortex and hippocampus revealed by single-cell RNA-seq. Science 347: 1138-1142 Doi 10.1126/science.aaa1934

6 Zhang Y, Chen K, Sloan SA, Bennett ML, Scholze AR, O'Keeffe S, Phatnani HP, Guarnieri P, Caneda C, Ruderisch Net al (2014) An RNA-sequencing transcriptome and splicing database of glia, neurons, and vascular cells of the cerebral cortex. The Journal of neuroscience : the official journal of the Society for Neuroscience 34: 11929-11947 Doi 10.1523/JNEUROSCI.1860-14.2014
